# Data-driven exploration of new pressure-induced superconductivity in PbBi_2_Te_4_

**DOI:** 10.1080/14686996.2018.1548885

**Published:** 2018-11-16

**Authors:** Ryo Matsumoto, Zhufeng Hou, Masanori Nagao, Shintaro Adachi, Hiroshi Hara, Hiromi Tanaka, Kazuki Nakamura, Ryo Murakami, Sayaka Yamamoto, Hiroyuki Takeya, Tetsuo Irifune, Kiyoyuki Terakura, Yoshihiko Takano

**Affiliations:** a International Center for Materials Nanoarchitectonics (MANA), National Institute for Materials Science, Ibaraki, Japan; b Graduate School of Pure and Applied Sciences, University of Tsukuba, Ibaraki, Japan; c Research and Services Division of Materials Data and Integrated System (MaDIS), National Institute for Materials Science, Ibaraki, Japan; d Center for Crystal Science and Technology, University of Yamanashi, Yamanashi, Japan; e Yonago College, National Institute of Technology, Tottori, Japan; f Geodynamics Research Center, Ehime University, Ehime, Japan; g Center for Materials research by Information Integration (CMI^2^), National Institute for Materials Science, Ibaraki, Japan

**Keywords:** Data-driven, superconductivity, high-pressure, 60 New topics / Others, 404 Materials informatics / Genomics, 302 Crystallization / Heat treatment / Crystal growth, 210 Thermoelectronics / Thermal transport / insulators

## Abstract

Candidate compounds for new thermoelectric and superconducting materials, which have narrow band gap and flat bands near band edges, were exhaustively searched by the high-throughput first-principles calculation from an inorganic materials database named AtomWork. We focused on PbBi_2_Te_4_ which has the similar electronic band structure and the same crystal structure with those of a pressure-induced superconductor SnBi_2_Se_4_ explored by the same data-driven approach. The PbBi_2_Te_4_ was successfully synthesized as single crystals using a melt and slow cooling method. The core level X-ray photoelectron spectroscopy analysis revealed Pb^2+^, Bi^3+^ and Te^2-^ valence states in PbBi_2_Te_4_. The thermoelectric properties of the PbBi_2_Te_4_ sample were measured at ambient pressure and the electrical resistance was also evaluated under high pressure using a diamond anvil cell with boron-doped diamond electrodes. The resistance decreased with increasing of the pressure, and pressure-induced superconducting transitions were discovered at 2.5 K under 10 GPa. The maximum superconducting transition temperature increased up to 8.4 K at 21.7 GPa. The data-driven approach shows promising power to accelerate the discovery of new thermoelectric and superconducting materials.

## GRAPHICAL ABSTRACT

**Figure:**
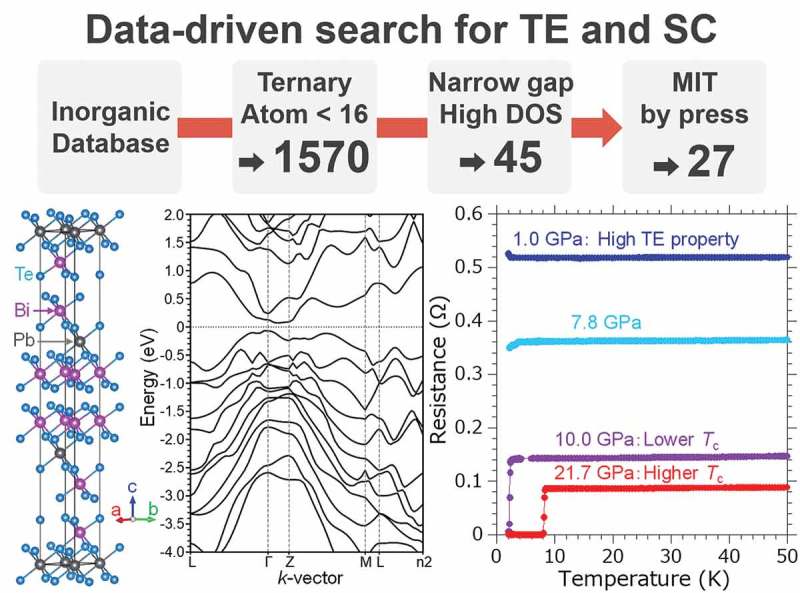

## 1. Introduction

A data-driven approach based on high-throughput computation has recently been applied successfully to exploration of new functional materials such as battery materials, thermoelectric materials, superconductors, and so on. Once proper target quantities are selected, this approach may be more efficient than or at least complementary to traditional carpet-bombing type experiments based on experiences and inspirations of researchers [1–5]. We have reported a case study of the data-driven approach thorough a discovery of pressure-induced superconductivity in a compound SnBi_2_Se_4_ selected by the high-throughput screening [6]. In this particular screening, the candidate compounds were explored according to a guideline that is characterized by specific band structures of ‘flat band’ near the Fermi level, such as multivalley [7], pudding mold [8], and topological-type [9] structures. If such kinds of flat band are realized near the Fermi level, thermoelectric properties with high electrical conductivity and Seebeck coefficient would be enhanced [8,10]. If the flat band crosses the Fermi level, superconductivity would be realized due to high density of states (DOS) [11–13]. Experimentally, a single crystal of SnBi_2_Se_4_ exhibited an insulator-to-metal transition under 11 GPa [6], in a good agreement with the theoretical prediction. Moreover, a pressure-induced superconductivity was observed with maximum superconducting transition temperature (*T*_c_) of 5.4 K under 63 GPa in accordance with our scenario. That work serves as a case study of the important ﬁrst step for next-generation data-driven material science.

In the aforementioned data-driven approach, a high thermoelectric performance in SnBi_2_Se_4_ is expected under high pressure around its insulator to metal transition, since the band gap decreases by the applied pressure, and then the flat band approaches the Fermi level. If a certain compound with the same crystal structure and similar band shape has narrower band gap than that of SnBi_2_Se_4_, it will show superior thermoelectric property even at ambient pressure. Furthermore, it could be expected that superconductivity may appear at much lower pressure, compared with SnBi_2_Se_4_.

Based on these considerations, we focused on PbBi_2_Te_4_ as a target compound because it has same crystal structure and similar band structure with the band gap narrower than ~200 meV of SnBi_2_Se_4_. Superior thermoelectric properties at ambient pressure and the superconductivity under lower pressure could be expected in PbBi_2_Te_4_, compared with SnBi_2_Se_4_. In this study, we successfully synthesized the sample of PbBi_2_Te_4_ in a single crystal. The crystal structure, compositional ratio, and valence states of the PbBi_2_Te_4_ single crystal were analyzed by the powder X-ray diffraction (XRD), an energy dispersive X-ray spectrometry (EDX) and an X-ray photoelectron spectroscopy (XPS), respectively. The thermoelectric properties were measured at ambient pressure. The resistivity of the obtained sample was evaluated under high pressure using a diamond anvil cell (DAC) with boron-doped diamond electrodes [14-17].

## 2. Screening procedures in high-throughput first-principles calculations

One thousand five hundred seventy candidates were listed from the inorganic material database named AtomWork [18], based on the following restriction: abundant and nontoxic or less toxic constituent elements, and the number of atoms being less than 16 per primitive unit cell. The candidates were narrowed down by using the restriction of a narrow band gap and high DOS near the Fermi level. By this screening, the number of candidate compounds was reduced to 45. Finally, we checked whether the band gap decreases (or even the metallic behavior appears) under pressure of 10 GPa, and screened out 27 promising compounds. Through the above screening procedures, PbBi_2_Te_4_ was chosen as a candidate for new thermoelectric and superconducting materials. The details of our screening scheme in the high-throughput first-principles calculations were given in our previous paper [6].

**Figure 1.**
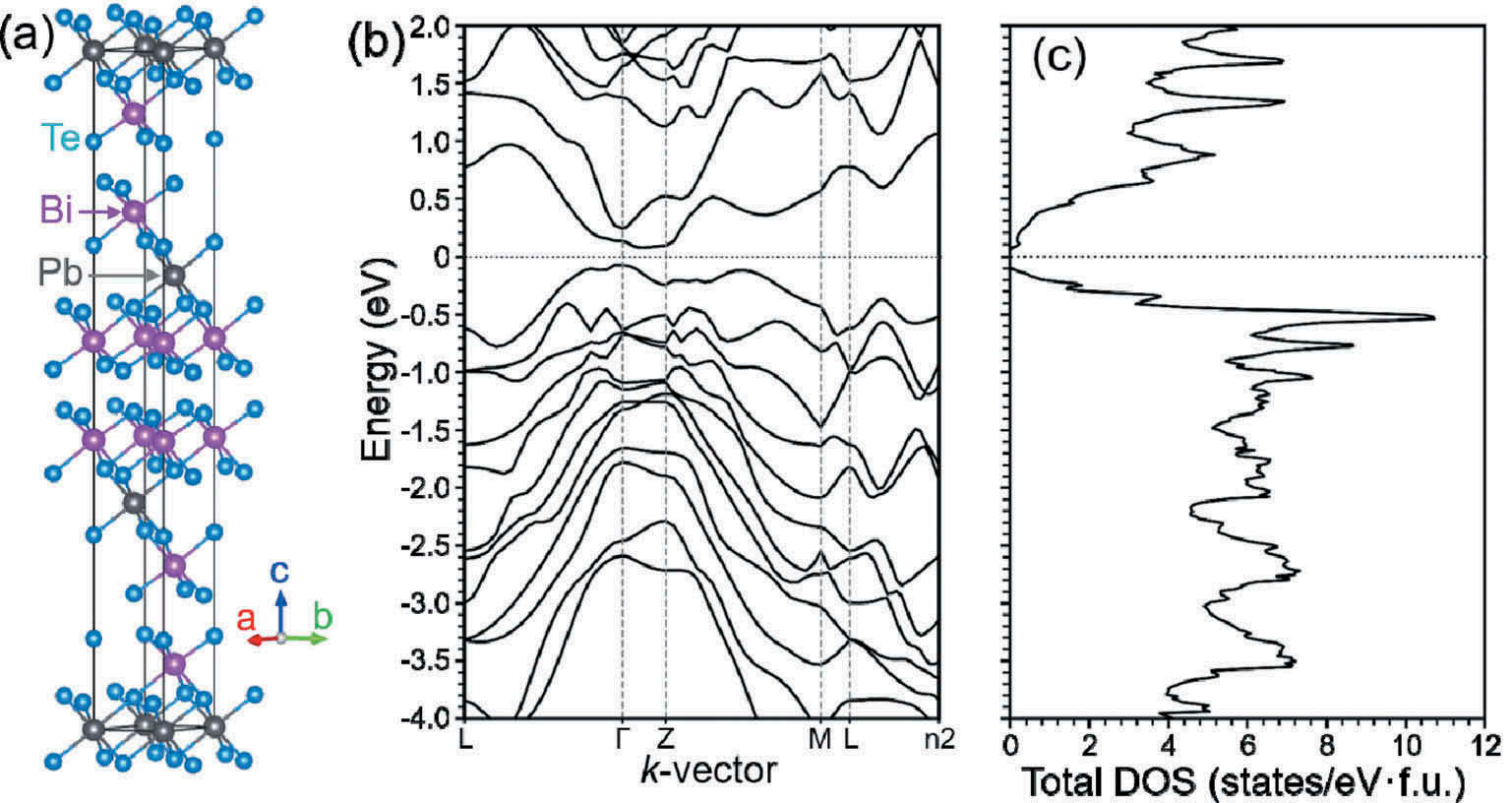
(a) Crystal structure, (b) band structure and (c) total density of states (DOS) of PbBi_2_Te_4_ obtained by the generalized gradient approximation with spin-orbit coupling.

Figure 1 shows (a) the crystal structure of PbBi_2_Te_4_ with trigonal *R-3m* structure depicted by VESTA [19], (b) the band structure and (c) the total DOS of PbBi_2_Te_4_ obtained by the generalized gradient approximation with spin-orbit coupling. We can see that the band edges of PbBi_2_Te_4_ show flat shape near the Fermi level. This feature is quite similar with that of SnBi_2_Se_4_. Additionally, the band gap of 101 meV in PbBi_2_Te_4_ is less than half of 208 meV in SnBi_2_Se_4_. The feature in the band structure of PbBi_2_Te_4_ at ambient pressure is similar to that of SnBi_2_Se_4_ under pressure of 5–10 GPa [6]. We could expect superior thermoelectric and superconducting properties under relatively low pressure for PbBi_2_Te_4_.

## 3. Experimental procedures

### 3.1. Sample synthesis

Single crystals of PbBi_2_Te_4_ were grown by a melt and slow-cooling method. Starting materials of Pb grains, Bi grains, and Te chips were put into an evacuated quartz tube in the stoichiometric composition of PbBi_2_Te_4_. The ampoule was heated at 1000 °C for 1 h, and then slowly cooled down to 800 °C within 20 h in the furnace. After keeping the temperature for 5 h, the ampoule was cooled down to room temperature. The obtained samples were ground and loaded into an evacuated quartz tube again. The sample was heated at 500 °C for 50 h for homogenization of PbBi_2_Te_4_ phase.

### 3.2. Characterization

The crystal structure of the obtained PbBi_2_Te_4_ samples was investigated by the powder XRD using the Mini Flex 600 setup (Rigaku) and Cu K*α* radiation. The lattice constants were refined using the Conograph software (High Energy Accelerator Research Organization, Japan) [20]. The chemical composition of the sample was evaluated by an EDX analysis using the JSM-6010LA microscope (JEOL). The valence state was estimated by the core level XPS analysis using AXIS-ULTRA DLD (Shimadzu/Kratos) with monochromatic Al K*α* X-ray radiation (*hν* = 1486.6 eV), operating under a pressure of the order of 10^−9^ Torr. The samples were cleaved using Scotch tape in a vacuum of approximately 10^−7^ Torr. The analyzed area was approximately 1 × 1 mm^2^. The binding energy scale was established by referencing the C 1s value of adventitious carbon. The background signals were subtracted by using the active Shirley method implemented in COMPRO software (Surface Analysis Society of Japan, Japan) [21]. The photoelectron peaks were analyzed by the pseudo-Voigt functions peak ﬁtting.

### 3.3. Transport measurements

**Figure 2.**
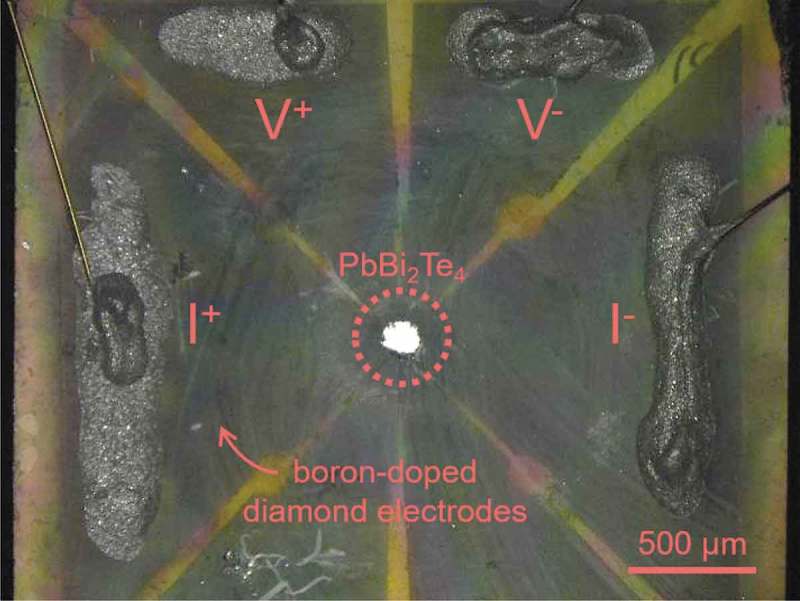
Optical image of the sample space of DAC with boron-doped diamond electrodes.

Thermoelectric properties, including the electrical resistivity, Seebeck coefficient, and thermal conductivity, were measured by using the thermal transport option (TTO) of physical property measurement system (PPMS/Quantum Design) under ambient pressure from 300 K to 2 K. The power factor and figure of merit were evaluated from the obtained parameters. Resistance measurements of PbBi_2_Te_4_ single crystal under high pressure were performed using an originally designed DAC with boron-doped diamond electrodes [14–17]. Figure 2 shows an optical image of the sample space of our DAC. The sample was placed at the center of the bottom anvil where the boron-doped diamond electrodes were fabricated. The undoped diamond insulating layer covers the surface of the bottom anvil except for the sample space and electrical terminal. The details of the cell configuration were described in the literature [16]. Cubic boron nitride powder with ruby manometer was used as a pressure-transmitting medium. The applied pressure values were estimated by the fluorescence from ruby [22] and the Raman spectrum from the culet of top diamond anvil [23] by an inVia Raman Microscope (RENISHAW).

## 4. Results and discussion

### 4.1. Crystal structure, composition and valence state

**Figure 3.**
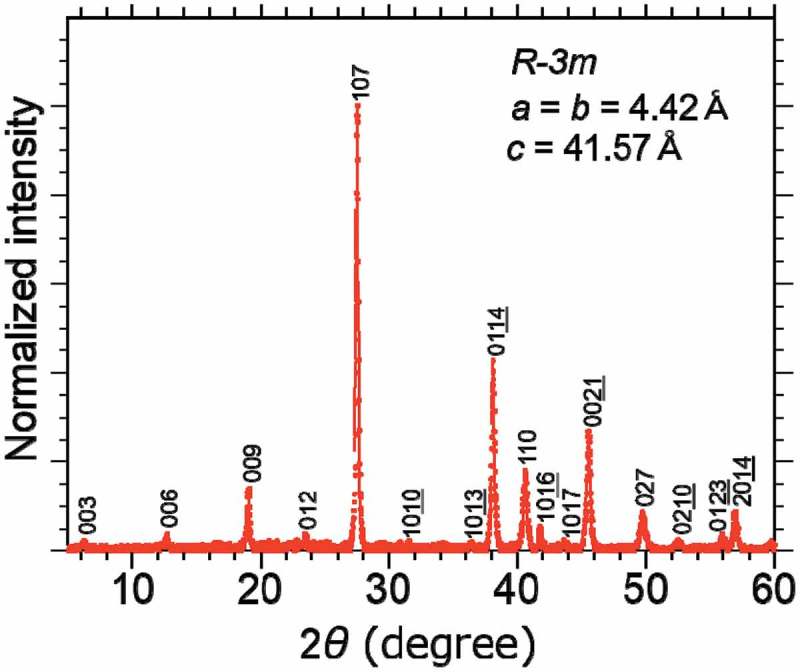
Powder XRD pattern of pulverized PbBi_2_Te_4_ single crystal.

Figure 3 shows a powder XRD pattern of the pulverized PbBi_2_Te_4_ single crystal. All observed peaks were well indexed to trigonal *R-3m* structure with lattice constants of *a* = *b* = 4.42 Å and *c* = 41.57 Å, without any impurity peaks. Here we note that if the sample is synthesized without the annealing process at 500 °C for 50 h which is described in the experimental section, the PbBi_4_Te_7_ contamination appears. EDX analysis of the obtained single crystal yields the composition Pb_0.9_Bi_2_Te_3.8_ as normalized by Bi, indicating Pb deficiency in the sample. The deficient nature is consistent with a related compound SnBi_2_Se_4_ [24].

**Figure 4.**
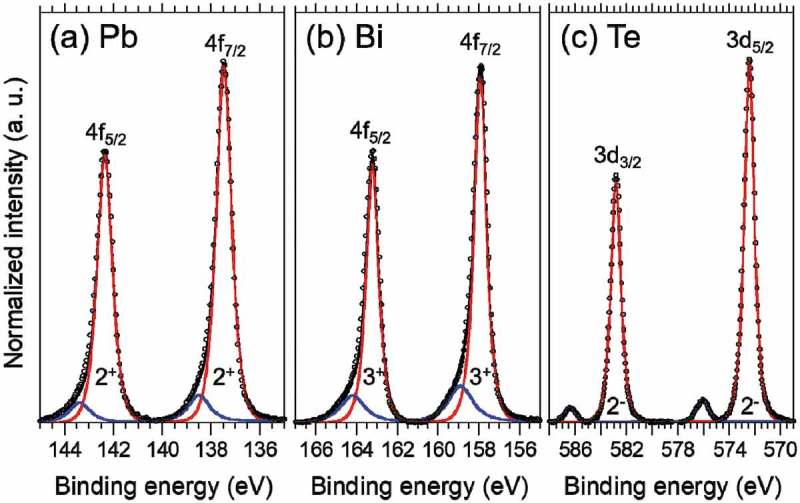
High-resolution XPS spectra of (a) Pb 4f, (b) Bi 4f, and (c) Te 3d core levels in PbBi_2_Te_4_ single crystal.

The valence states of Pb, Bi and Te in PbBi_2_Te_4_ were investigated by XPS. Figure 4(a) shows a Pb 4f core-level spectrum of PbBi_2_Te_4_. There are two main peaks at 142.4 eV and 137.5 eV corresponding to Pb 4f_5/2_ and 4f_7/2_ with the valence state of Pb^2+^ [25]. Figure 4(b) shows a Bi 4f core-level spectrum. The Bi 4f photoemission is split into two peaks, one at around 157.8 eV attributed to Bi 4f_7/2_ and the other at around 163.1 eV attributed to Bi 4f_5/2_ [26]. These main peak positions are corresponding to that of Bi^3+^ valence state. The Te 3d region had two groups of peaks as shown in Figure 4(c), which were observed at 582.8 eV and 572.4 eV, indicating the existence of Te^2-^, 586.5 eV and 576.1 eV of Te^4+^ due to a surface oxide layer [27]. These Pb^2+^, Bi^3+^ and Te^2-^ are consistent with the formal charge valence of PbBi_2_Te_4_. The Pb 4f and Bi 4f spectra contain small peaks at the higher binding energy region which may be due to the surface oxidization or the asymmetric feature of the main peaks [28]. If the asymmetry can be fitted with a Doniach-Sunjic line shape [29], it means the sample is metallic and has high DOS near the Fermi level [30]. Figure S1 in the supplemental materials shows the outcome of peak fitting using the Doniach-Sunjic line shaped pseudo-Voigt functions. The asymmetric parameter *α* was 0.12 and 0.09 in Bi 4f and Pb 4f, respectively. Although the peak shape is consistent with the flat band model, further investigation is necessary to determine the reason of its asymmetry.

### 4.2. Thermoelectric properties

**Figure 5.**
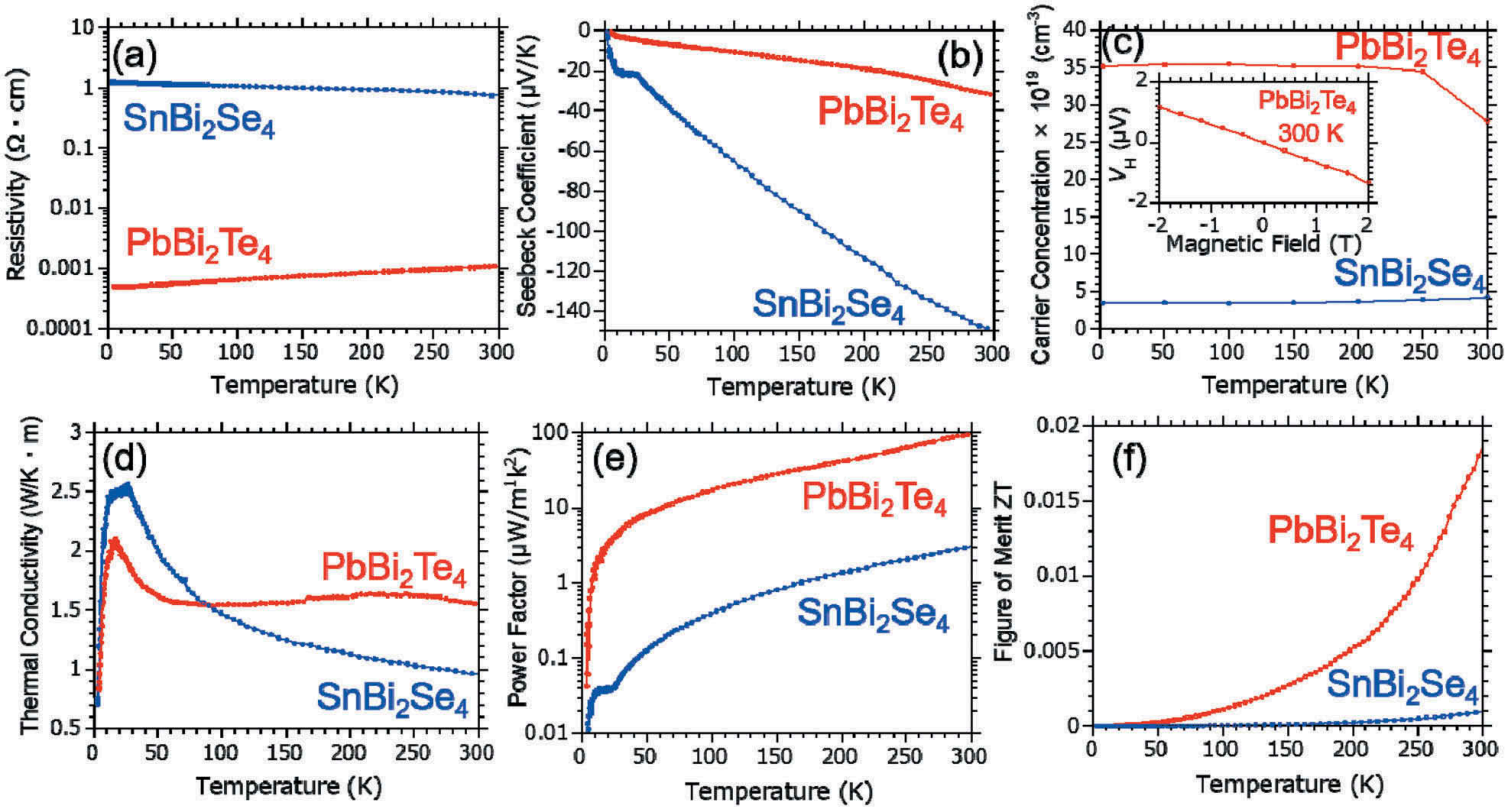
Temperature dependence of thermoelectric properties in PbBi_2_Te_4_ and SnBi_2_Se_4_ under ambient pressure. (a) resistivity, (b) Seebeck coefficient, (c) carrier concentration (inset is a magnetic field dependence of Hall voltage at room temperature), (d) thermal conductivity, (e) power factor, and (f) figure of merit ZT.

Figure 5 shows temperature dependence of the thermoelectric properties including (a) electrical resistivity, (b) Seebeck coefficient, (c) carrier concentration, and (d) thermal conductivity under ambient pressure for PbBi_2_Te_4_ (red curves). The results of SnBi_2_Se_4_ (blue curves) are also shown for comparison. PbBi_2_Te_4_ shows negative slope of resistivity toward lower temperature, namely metallic behavior. The absolute value of resistivity is much smaller than that of SnBi_2_Se_4_ [6]. The negative Seebeck coefficient and negative slope of the Hall voltage as a function of applied magnetic field indicate n-type nature of the sample, which may be caused by the excess Bi in the crystal. If the carrier type is tuned from n-type to p-type, the thermoelectric property could be enhanced because the valence band edge provides higher DOS near the Fermi level. A high pressure application, elemental substitution, or electric double layer transistor gating are effective for such kind of band tuning. Although the absolute value of the Seebeck coefficient in PbBi_2_Te_4_ is smaller than that of SnBi_2_Se_4_, the thermal conductivity is almost same [6]. Consequently, the thermoelectric properties of the power factor and figure of merit were dramatically increased in PbBi_2_Te_4_ compared to SnBi_2_Se_4_. The highest values of the power factor ~100 μWm^−1^ K^−2^ and figure of merit ~ 0.02 were obtained at 300 K. This tendency of high thermoelectric performance would be originated from the flat band with the small band gap, which is equivalent to the electronic state in SnBi_2_Se_4_ under high pressure [6].

### 4.3. In situ resistivity measurement under high pressure

**Figure 6.**
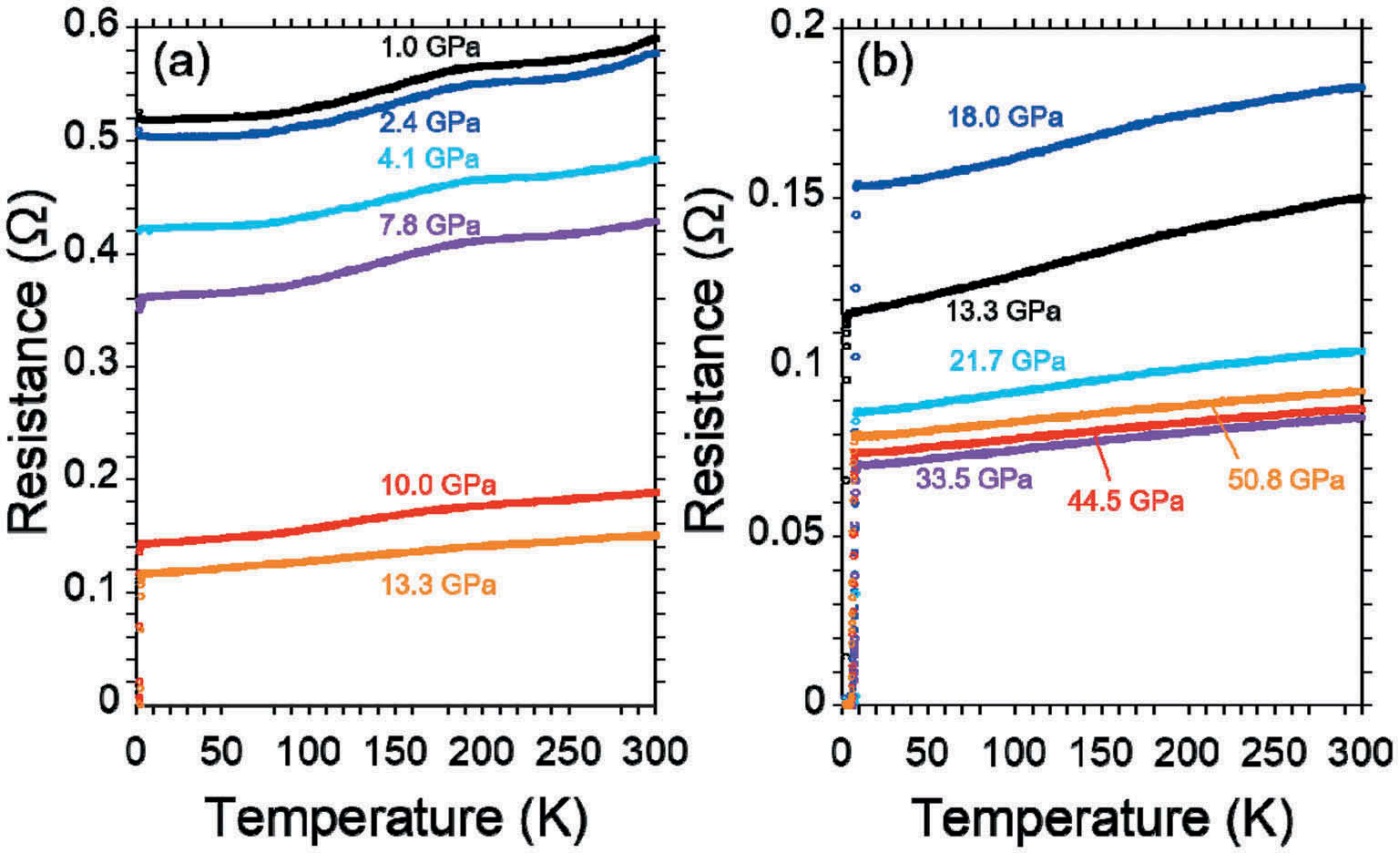
Temperature dependence of resistance in PbBi_2_Te_4_ under various pressures, (a) 1.0–13.3 GPa, (b) 13.3–50.8 GPa.

Figure 6(a) shows a temperature dependence of resistance for PbBi_2_Te_4_ under various pressures from 1.0 GPa to 13.3 GPa. The sample has already exhibited metallic behavior under ambient pressure as shown in Figure 5(a). The resistance at 1.0 GPa also shows metallic behavior but with a small hump around 200 K. The resistance and the intensity of hump decreased with the increase of the applied pressure. A pressure-induced superconductivity with clear zero resistance was observed under 10 GPa. This critical pressure of the superconductivity is almost half of 20.2 GPa in SnBi_2_Se_4_. In this region, the maximum onset transition temperature (*T*_c_^o^ ^nset^) and zero-resistance temperature (*T*_c_^zero^) were 3.4 K and 2.4 K under 13.3 GPa, respectively.

**Figure 7.**
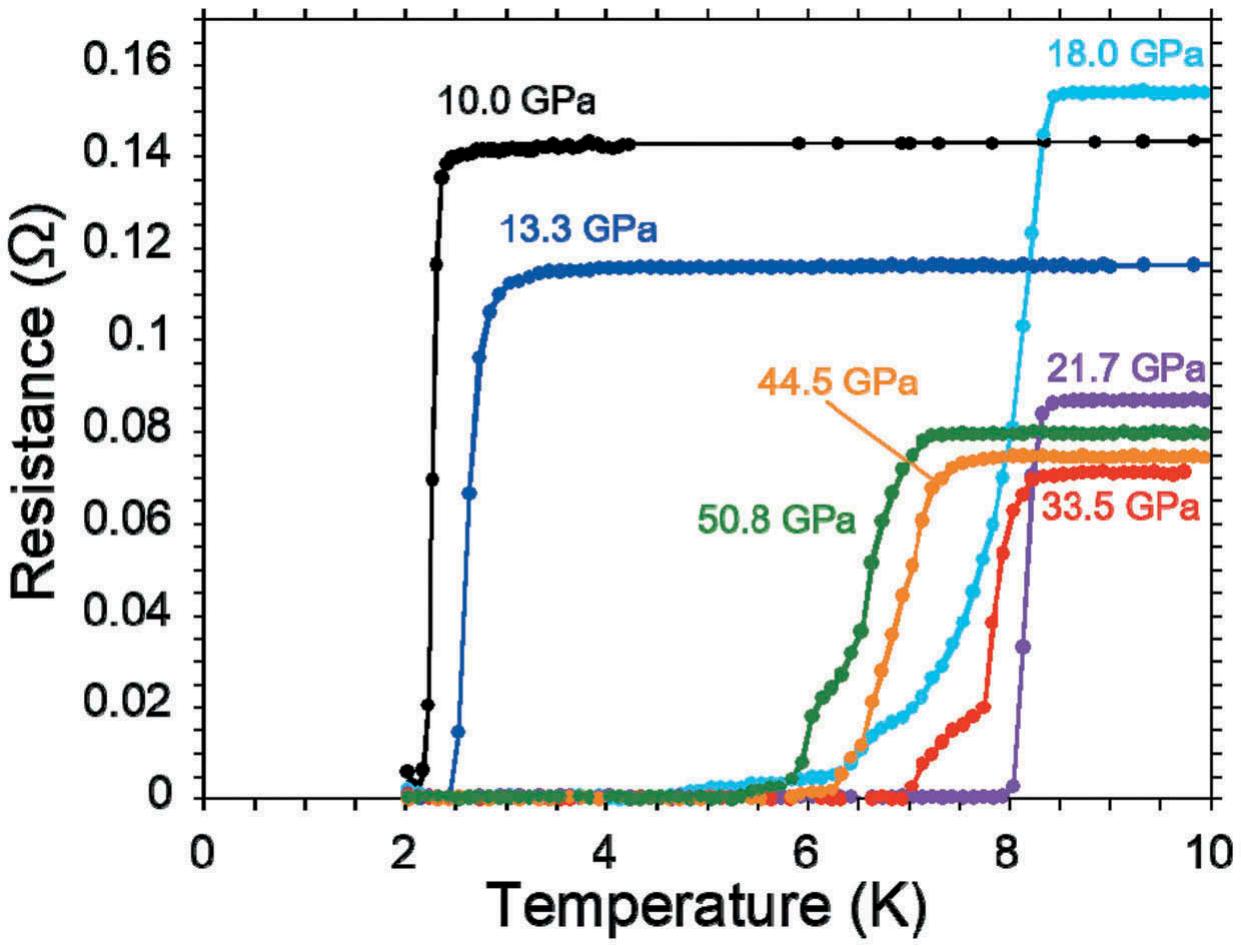
Temperature dependence of resistance around superconducting transitions in PbBi_2_Te_4_ under various pressures.

The *T*_c_ of PbBi_2_Te_4_ suddenly jumped up with the application of further pressure as same as SnBi_2_Se_4_. A temperature dependence of resistance from 13.3 GPa to 50.8 GPa is shown in Figure 6(b). The *T*_c_^onset^ was enhanced from 3.4 K under 13.3 GPa to 8.1 K under 18.0 GPa. In the higher pressure region, the maximum *T*_c_^onset^ and *T*_c_^zero^ were 8.4 K and 7.9 K under 21.7 GPa, respectively. The temperature dependences of resistance around the superconducting transitions are summarized in Figure 7. Indeed, the tendency of the *T*_c_ increases is quite similar to that of SnBi_2_Se_4_ [6]. Both the critical pressures for the lower and higher *T*_c_ phases under ~10 GPa and ~20 GPa in PbBi_2_Te_4_ are almost half of ~20 GPa and ~40 GPa in the SnBi_2_Se_4_, respectively, due to the band gap difference. The higher *T*_c_ in PbBi_2_Te_4_ compared with that in SnBi_2_Se_4_ would be originated from the higher DOS near the Fermi level because the higher pressure application decreases the DOS due to an increase of bandwidth.

**Figure 8.**
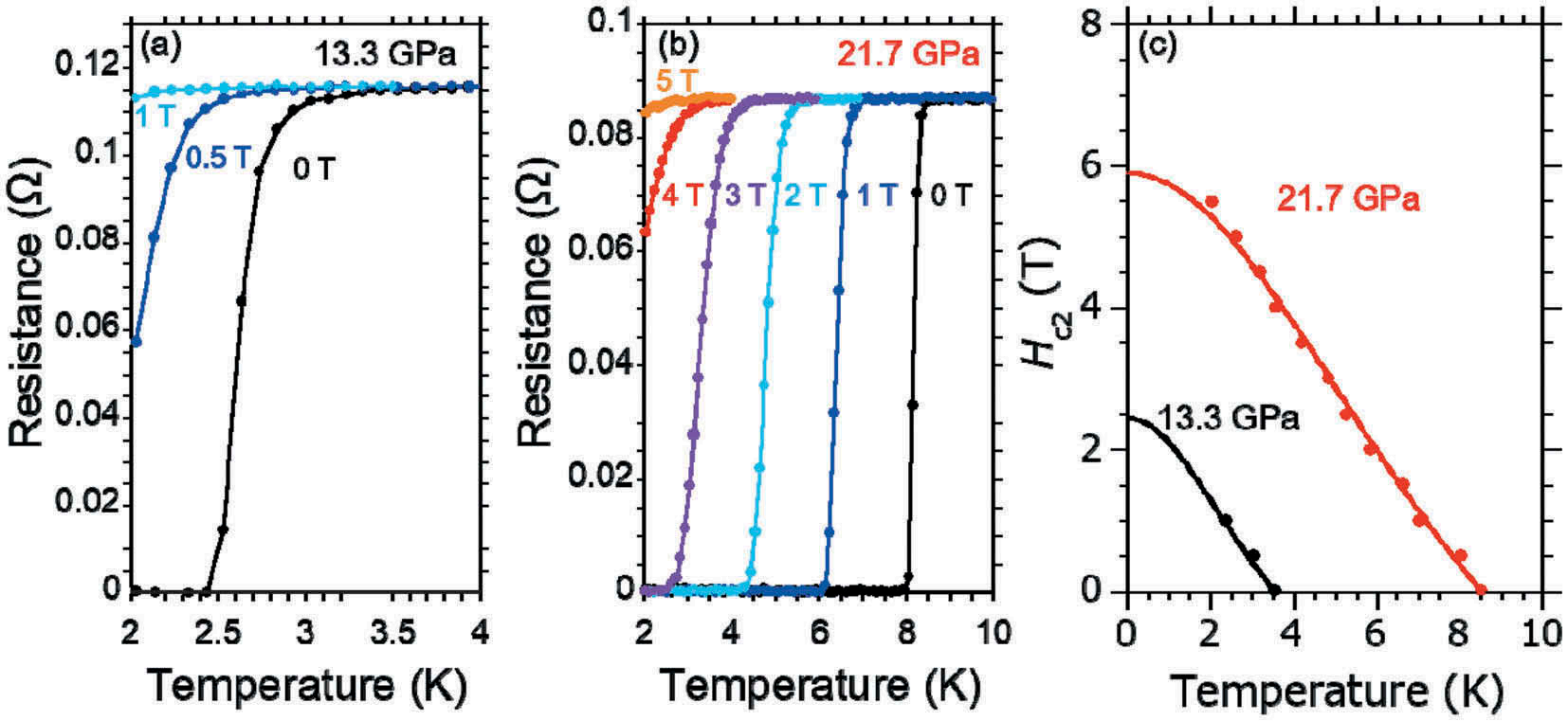
Temperature dependence of resistance of PbBi_2_Te_4_ in specified magnetic field, under pressure of (a) 13.3 GPa or (b) 21.7 GPa. (c) Temperature dependence of *H*_c2_^//*ab*^ values at 13.3 GPa and 21.7 GPa.

Figure 8 shows temperature dependence of resistance in various magnetic fields under (a) 13.3 GPa, (b) 21.7 GPa. Upper critical field *H*_c2_^//*ab*^(0) values were estimated from the Werthamer-Helfand-Hohenberg (WHH) approximation [31] for the Type II superconductor in a dirty limit. A temperature dependence of *H*_c2_^//*ab*^ values is shown in Figure 8(c). The *H*_c2_^//*ab*^(0) were 2.4 T under 13.3 GPa and 5.9 T under 21.7 GPa.

**Figure 9.**
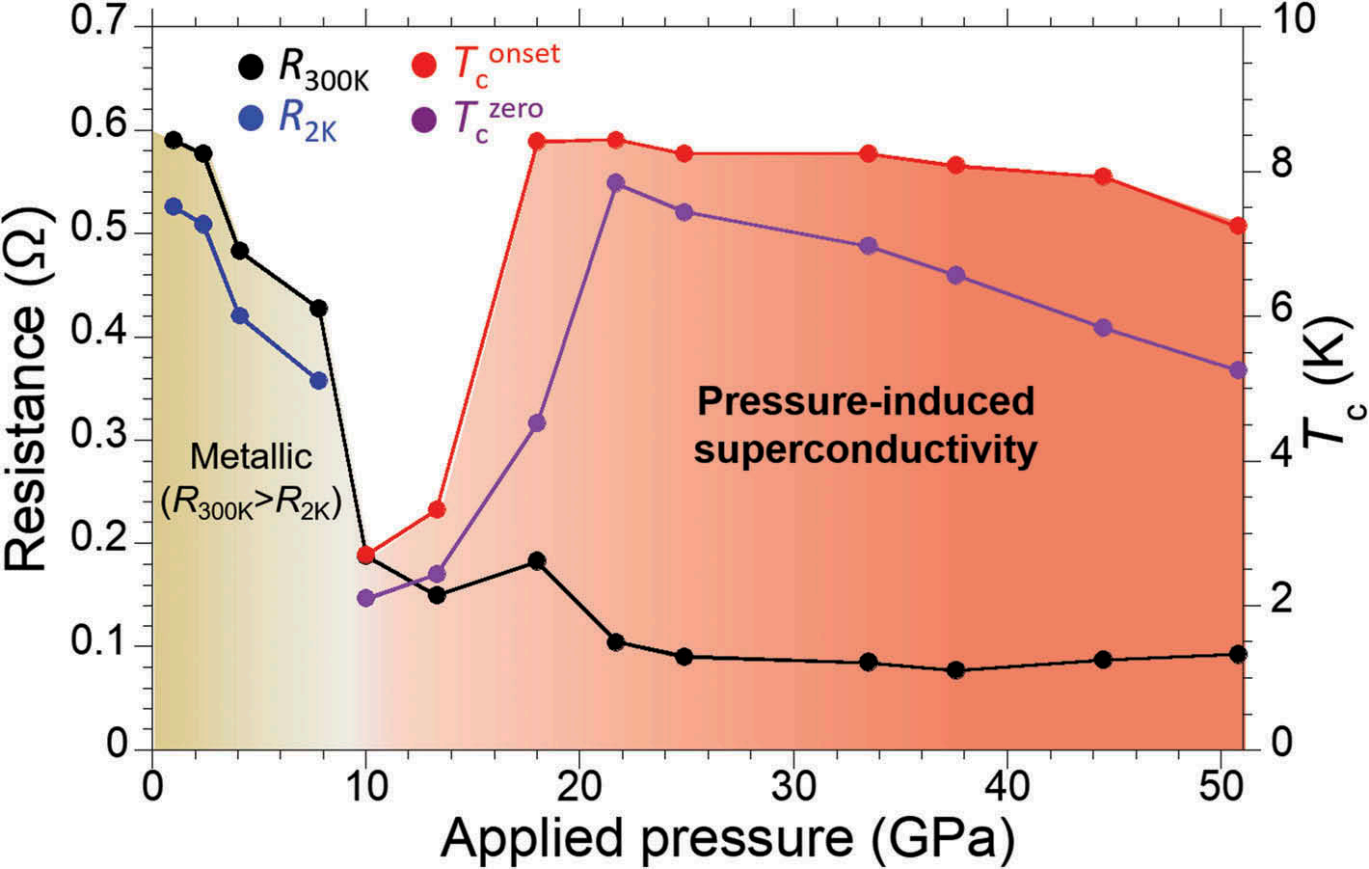
Resistance-pressure phase diagram of PbBi_2_Te_4._

Figure 9 shows a resistance-pressure phase diagram of PbBi_2_Te_4_ single crystal. The resistance of the sample is dramatically decreased by applying pressure. After that, the first superconducting phase was newly discovered under 10.0 GPa. In a higher pressure region above 18.0 GPa, we observed a *T*_c_ jump from 3.4 K to 8.4 K. The *T*_c_ jump indicates a possibility of appearance of second superconducting phase. Further experimental investigation is required to clear the origin of *T*_c_ jump, for example, analysis of pressure distribution in sample space, *in situ* XRD measurement under high pressures, and theoretical prediction of crystal structure under high pressure. This superconductivity survived up to at least 50.8 GPa. The *T*_c_ and *H*_c2_^//*ab*^(0) values were almost independent of the applied pressure.

## 5. Conclusion

Among 27 compounds suggested by the data-driven approach, we focused on PbBi_2_Te_4_ from the viewpoint of band similarity to the pressure-induced superconductor SnBi_2_Se_4_ which was also chosen by the data-driven approach. The PbBi_2_Te_4_ has similar flat band feature with a smaller band gap, compared to that of SnBi_2_Se_4_. The synthesized PbBi_2_Te_4_ single crystal exhibited better thermoelectric properties at ambient pressure and higher *T*_c_ value under high pressure. Especially, the superconductivity occurred under a pressure of approximately 10 GPa lower than ~ 20 GPa in SnBi_2_Se_4_, in accordance with our scenario. The present work presents a case study for the important first-step for the next generation data-driven materials science.
