# Supplementary figures and images for: Data-driven exploration of new pressure-induced superconductivity in PbBi2Te4
Source: Sci Technol Adv Mater. 2018 Nov 16;19(1):909–16. doi: 10.1080/14686996.2018.1548885 (PMC6319466; doi:10.1080/14686996.2018.1548885)

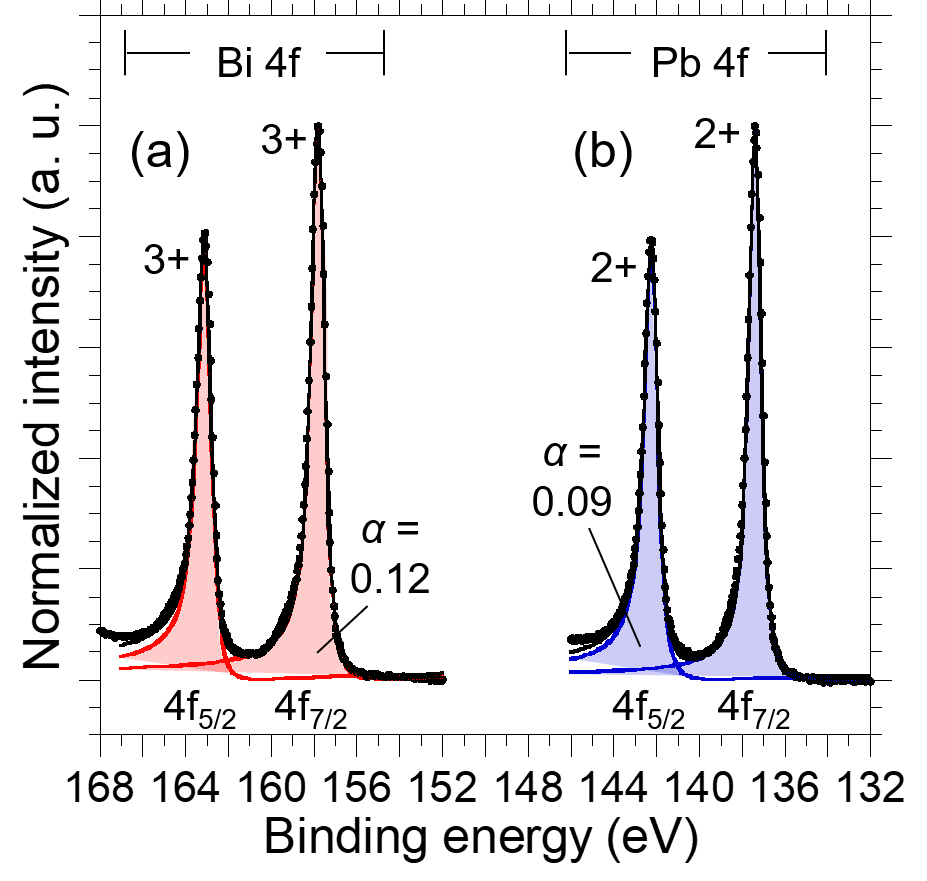

Supplement: Supplemental Material [file TSTA_A_1548885_SM3292.tif]
